# Evaluation of urine dipstick tests in experimental porcine urinary tract infection with uropathogenic *Escherichia coli*

**DOI:** 10.1038/s41598-023-39239-7

**Published:** 2023-07-31

**Authors:** Kristian Stærk, Louise Kruse Jensen, Thomas Emil Andersen

**Affiliations:** 1 Department of Clinical Microbiology, Odense University Hospital, Odense, Denmark; 2 Research Unit of Clinical Microbiology, University of Southern Denmark, Odense, Denmark; 3 Pathobiological Sciences, University of Copenhagen, Copenhagen, Denmark

**Keywords:** Infectious-disease diagnostics, Urinary tract infection

## Abstract

Urinary tract infection is a common disease in pigs and a major reason for sows to be culled. The disease, however, is difficult to diagnose due to lack of distinct clinical signs in the animals. We evaluated the diagnostic value of two commercial urine dipstick tests in 10 pigs using an experimental model of *Escherichia coli* urinary tract infection. Urine collected at baseline and 48 h after inoculation were analyzed. We show that dipstick tests positive of blood, leucocytes and particularly nitrite are very specific for *E. coli* UTI with a 100% positive predictive value.

## Introduction

Urinary tract infection (UTI) is a common disease in pigs and constitutes an economic burden in swine production as the disease may influence reproductive performance or result in culling of the animal^1,2^. To maintain production and improve animal welfare, early treatment is critical as delayed intervention may increase the risk of pyelonephritis or treatment-failure^2^. However, diagnosing UTI in commercial herds is not easily performed for the following reasons: (i) pigs do not show clear clinical signs of the disease, thus making it difficult to identify suspected subjects; (ii) the diagnostic gold standard is a positive urine culture which is time-consuming, demanding at least 48 h of laboratory work to identify the pathogen and associated antibiotic susceptibility^3^. Urine dipstick tests are a quick and inexpensive method that may facilitate early identification of infected animals. Although urine dipstick tests have been suggested to offer early warning of UTI in pigs, these studies, as well as most studies of porcine UTI, are based on urine specimens and tissue collected from animals with spontaneous UTI or culled animals in abattoirs or rendering plants; hence, the analyzed samples originate from heterogenous cases of the disease or do not accurately represent biological specimens collected in live animals^1,4,5^. Furthermore, urine dipstick tests are developed for human use and are not routinely used in pigs. Consequently, the diagnostic sensitivity is not convincingly validated in these animals^1,3,6,7^. The aim of this study was to evaluate the diagnostic value of commercial urine dipstick tests for diagnosing UTI in pigs infected with uropathogenic *Escherichia coli* (UPEC) under controlled experimental conditions. The study was facilitated by recent advances in experimental porcine models of UTI^8–12^.

## Materials and methods

### Animals and housing

Ten 7-week old female pigs (Danish Landrace x Danish Yorkshire) were purchased from a local vendor (Kokkenborg ApS) and housed in the animal facility of the Biomedical Laboratory, University of Southern Denmark. The animals were kept in communal enclosures with sawdust bedding and fed a standard diet with free access to water. Enrichment was provided in the form of toys, music, and daily human interaction. The experiments were conducted after 7 weeks housing when the pigs had reached a mean weight of 72.7 kg (SD:6.5). The animals were attended at least twice daily and monitored for physical activity and food consumption at every inspection and weighed at least weekly.

### Anesthesia

Pigs were premedicated with a mixture of 1 vial of dry matter Zolazepam/tiletamine (Zoletil 50 Vet., Virbac Danmark) dissolved in 6.45 ml Xylazin (Sedaxylan Vet., 20 mg·mL^−1^, Dechra Veterinary Products), 1.25 ml Ketamine (Ketaminol Vet., 100 mg·mL^−1^, MSD Animal Health), 2 mL Butorphanol (Butomidor Vet., 10 mg·mL^−1^, Salfarm Danmark) and 2 ml Methadone (Insistor Vet., 10 mg·mL^−1^, Salfarm Danmark). Each pig was administered an intramuscular injection of 1.3 mL·10^–1^ kg bodyweight. The pigs were moved to the operating bed when muscular reflexes were absent.

### Bacteria

We used the uropathogenic *E. coli* isolate UTI89 which was originally isolated from a human cystitis patient. An overnight plate colony was incubated 21 h in lysogeny broth (LB) and from here 100 uL were subsequently cultured in fresh LB for another 24 h to optimize type-1 pili expression. On the day of the infection, the broth was centrifuged for 5 min at 5000*g*. The pellet was resuspended in saline and adjusted to an optical density of 1.0 at 600 nm, yielding a bacterial concentration of approximately 1·10^9^ colony forming units (CFU)·mL^−1^. This suspension was serial diluted to reach final inoculum concentrations of 1·10^2^ CFU·mL^−1^ (verified by plating on agar). The bacterial suspension was prepared immediately before inoculation.

### Infection protocol

**Figure 1:**
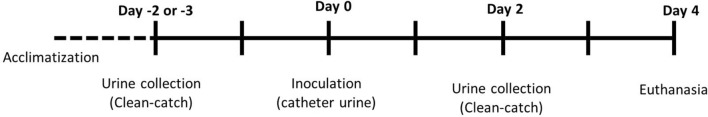
Timeline of the experimental protocol. Urine was collected non-invasively by clean-catch 2 or 3 days before infection and 2 days post infection and analyzed by urine dipstick tests. Urine was collected at day 0 by transurethral bladder catheter to confirm sterility of the urinary tract immediately before inoculation with uropathogenic *E. coli*.

A baseline urine sample was collected non-invasively by clean-catch two or three days prior to inoculation using a technique previously described^13^. On the day of inoculation, the pigs were inoculated with UPEC using an infection protocol based on Stærk et al.^9^. In short, sedated pigs were placed in dorsal recumbency and the urogenital area washed and disinfected with two rounds of 0.1% chlorhexidine. A 10 Fr foley type catheter (Rüsch) was placed aseptically, and a urine specimen collected to verify absence of bacteriuria. The bladder was then completely emptied to ensure equal basis of infection. In nine of the ten animals, a suspension of UPEC in 100 mL saline (10^2^ CFU·mL^−1^) was instilled into the bladder through the catheter to induce cystitis. One animal was mock-infected with sterile saline as control. The catheter was clamped for one hour before emptying the bladder completely and removing the catheter. Two days after inoculation, another urine sample was collected by clean-catch. Four days after inoculation, the pigs were euthanized with 0.35 mL·kg (bodyweight)^−1^ pentobarbital (400 mg·mL^−1^) and the death confirmed by auscultation. Whole bladders were removed post mortem within 5–10 min. From each bladder, two tissue specimens were punched out using a drive punch (Ø = 10 mm) and stored in 10% neutral buffered formalin for histopathological analysis (see below). Rectal temperature was measured at baseline, after 24 h and at termination. The timeline is summarized in Fig. 1.

### Urinalysis

Urine was cultured on blue agar plates (SSI diagnostica) and CFU manually counted after incubation at 35°C overnight. Two commercial urine dipstick tests were used in this study: (i) the Combur 7 Test (Roche) and (ii) Multistix 5 (Siemens). The urine specimens collected by clean-catch were tested by the dip stick tests according to the manufacturer’s instructions and always within 1 h after sampling. The results were interpreted by two individuals, and in the case of discrepancy, the test was re-evaluated until agreement on the result was reached. Both tests analyzed urine leucocytes, glucose, nitrite, protein and blood, while the Combur 7 Test also analyzed pH and ketones.

### Histopathology

Vesical specimens were placed in 10% neutral buffered formalin for 3–4 weeks. Following fixation, each biopsy was cut into two parts. Both halves of a biopsy were placed in cassettes with the cutting surface downwards, i.e. the site for sectioning and processed through graded concentrations of alcohol and embedded in paraffin wax. Tissue sections were cut (4 µm) and stained with hematoxylin and eosin (HE). HE stained sections were evaluated patho-morphologically with special focus on identifying an inflammatory response. Inflammation was semi-quantitatively scored as minor, moderate, or massive based on the quantity of inflammatory findings. The pathologist was blinded to the identification of the animals.

### Statistics

Statistical analyses were performed using GraphPad Prism version 9.3.1. Analysis of more than two groups were performed using One-way ANOVA with Tukey’s multiple comparisons test. For calculating positive and negative predictive values, the gold standard for UTI was determined as bacteriuria with associated inflammation. The contingency analysis was performed using Fisher’s exact test with Wilson-Brown test for predictive values.

### Ethics approval

All samples were collected as part of a tandem-project, thus determining the choice of animal breed, sex and number using the same series of pigs in a study approved by the Danish Animal Experiment Inspectorate, license number: 2021-15-0201-00821. The experiments were conducted according to the EU directive 2010/63/EU on the protection of animals used for scientific purposes and reported according to the ARRIVE guidelines.

## Results

### Infectious outcome

**Figure 2:**
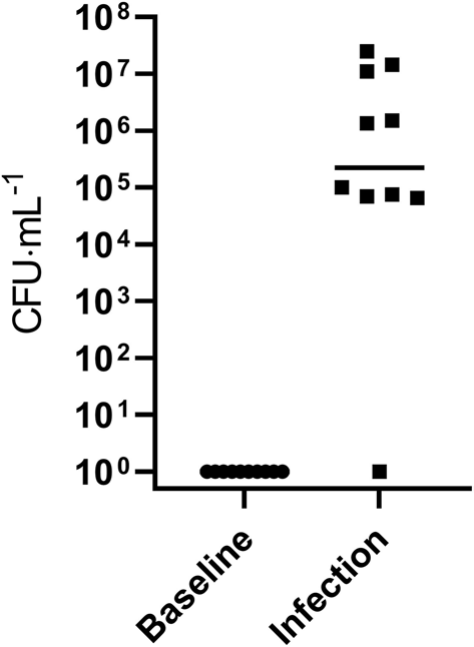
A urine sample collected prior to inoculation showed absence of bacteria in all animals. Two days after experimental inoculation with uropathogenic *Escherichia coli*, all animals (n = 9) developed bacteriuria > 10^4^ CFU·mL^−1^. One animal that was mock-infected did not develop bacteriuria (n = 1). Limits of detection was 10 CFU·mL^−1^. Horizontal bar indicates the mean.

The results showed no detectable bacteria at baseline confirming the absence of disease prior to inoculation (Fig. 2). After inoculation, all pigs developed a UTI determined by monoculture of *E. coli* above 10^4^ CFU·mL^−1^ and associated bladder inflammation (determined by histopathology) (Fig. 2). The control pig that was mock-infected did not develop bacteriuria. Mean temperatures (SD) at baseline, day 1 and at termination were 39.3 (0.23), 39.1 (0.22) and 39.2 (0.60), respectively. No changes in general behavior were observed. All tested parameters for both urine dipstick tests were negative in urine specimens collected at baseline. The results of the dipstick tests of urine samples from pigs during infection are summarized in Table 1. Using the Combur 7 test, nitrite, leucocytes, protein and blood were positive in 9, 3, 2 and 3 pigs (of 9), respectively. For the Multistix 5, the same numbers were 9, 0, 0 and 3, respectively. Macroscopic hematuria was never observed, even in pigs positive for blood. Glucose and ketones were always negative. No significant difference was observed in mean pH before (5.5, SD: 0.5) and after inoculation (5.6 SD: 0.7), determined by the Combur 7 test.

**Table 1:** Urine dipstick test outcome.

Variable	Positive % (n)		PPV	NPV	P
	Baseline (n = 10)	Infection (n = 9)			
Combur 7 Test					
Glucose	0 (0)	0 (0)	–	–	–
Ketones	0 (0)	0 (0)	–	–	–
Leukocytes	0 (0)	33 (3)	1.00	0.63	0.087
Nitrite	0 (0)	100 (9)	1.00	1.00	< 0.0001
Protein	0 (0)	22 (2)	1.00	0.59	0.21
Blood	0 (0)	33 (3)	1.00	0.63	0.087
Multistix 5					
Glucose	0 (0)	0 (0)	–	–	
Leukocytes	0 (0)	0 (0)	–	0.53	> 0.99
Nitrite	0 (0)	100 (9)	1.00	1.00	< 0.0001
Protein	0 (0)	0 (0)	–	0.53	> 0.99
Blood	0 (0)	33 (3)	1.00	0.63	0.087

*PPV* positive predictive value, *NPV* negative predictive value.

### Histopathological analysis

**Figure 3:**
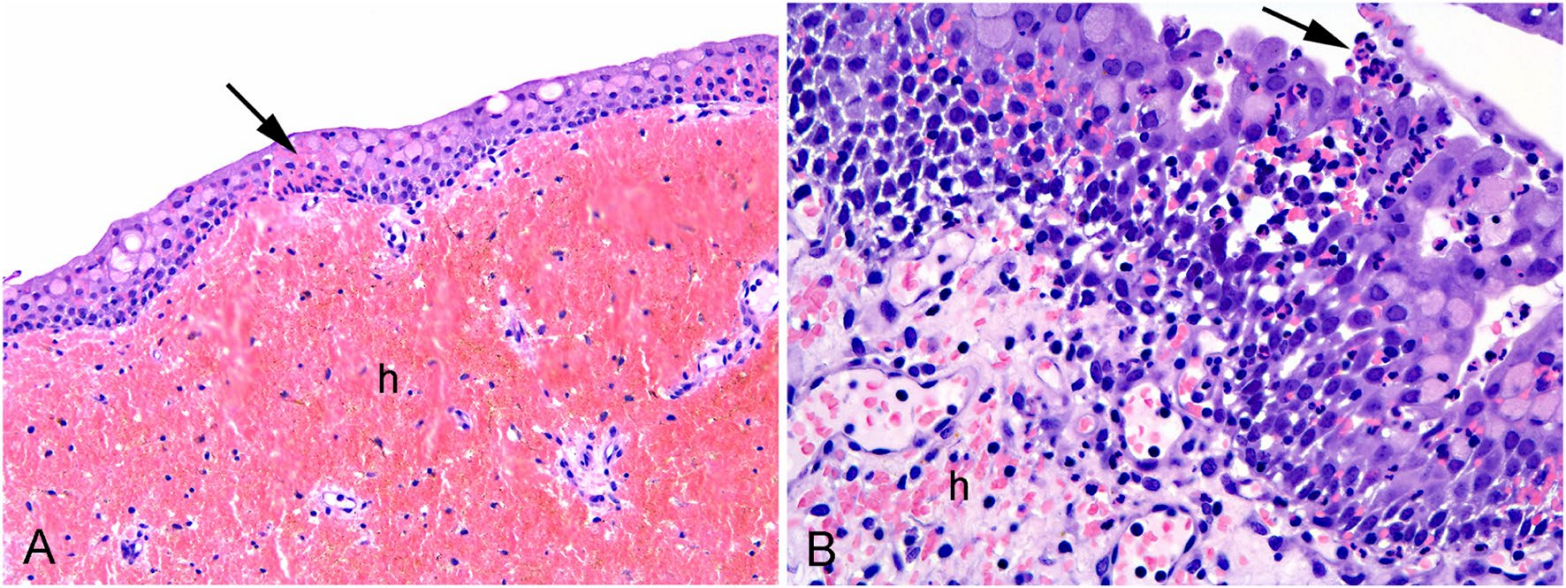
From pig no. 94494. (**A**) Massive bleeding (h) in lamina propia and within the epithelium (arrow). Vacuolization of the epithelium can be seen. (**B**) Focal massive neutrophil granulocyte infiltration in lamina propia and within the epithelium. The neutrophil morphology can clearly be recognized (arrow). Hemorrhage (h) is seen in lamina propia.

All inoculated animals showed inflammatory lesions and could therefore be diagnosed with an acute suppurative cystitis. The inflammatory changes included hemorrhage, edema, neutrophil infiltration, macrophage infiltration, and epithelial vacuolization (estimated to be a sub-lethal cell damage) and is summarized in Table 2. Inflammation was scored as mild in animals 94502 and 94683, moderate in animals 94591, 94616, and 94666, and massive in animals 94585, 94494, 94596, and 9440 (Fig. 3). Hemorrhage and inflammatory cellular infiltration were seen within lamina propria and occasionally also inside the epithelium, respectively. The cellular infiltration was concentrated in foci. No inflammatory signs were seen in the control animal (No. 94577).

**Table 2:** Histopathological outcome.

ID	Hemorrhage in lamina propria	Edema in lamina propria	Epithelial vacuolization	Neutrophile infiltration	Macrophage infiltration	Inflammation status
94666	+	+	−	+	+	Moderate
94616	+	+	+	+	+	Moderate
94494	+	+	+	+ (many)	+	Severe
94591	+ (massive)	+	+	+	+	Moderate
94502	+	+	+	+	+	Mild
94683	+	+	+	+	+	Mild
94585	+	+	+	+ (many)	+	Severe
94,596	+ (massive)	+	+	+ (many)	+	Severe
9440	+	+	+	+ (many)	+	Severe
94577	−	−	−	−	−	None

## Discussion

In this study we demonstrate the reliability of commercial urine dipstick tests for identifying UTI in pigs experimentally infected with *E. coli*. Using this approach, we were able to study porcine UTI under very controlled experimental conditions that has not previously been possible. We studied a group of pigs subjected to infection with an identical pathogen and collected urine specimens before and after infection from each animal.

We found that all animals developed bacteriuria in response to the experimental inoculation, as previously reported^9^. The bacteriuria was associated with tissue inflammation (determined by histopathology), congruent with symptomatic UTI (as opposed to asymptomatic colonization). The sensitivity, specificity, positive- and negative predictive values of nitrite were 100% in dipstick tests from both manufacturers, suggesting that nitrite may be an excellent indicator of *E. coli* UTI in suspected animals. The absence of nitrite in all baseline samples, i.e., from pigs without infection, suggests that a positive nitrate is specific for nitrite-producing microorganisms inside the bladder and that potential contaminants in urine specimens collected by clean-catch do not lead to false-positive results of this parameter. Based on these findings, nitrite is an important biomarker to consider when interpreting urine culture results that may be significantly influenced by contamination^2^. Hence, a positive nitrite may be used to differentiate UTI from contamination when urine cultures show *E. coli* colony counts of 10^3^–10^4^ CFU mL^−1^.

Although these results demonstrate nitrite as a useful biomarker of infection, the practical applicability of this parameter has two limitations: (i) although most uropathogens are nitrite-producers, *Enterococcus spp.* and other porcine uropathogens such as *Actinobaculum suis* are not; (ii) it is known from humans that nitrite may be produced by asymptomatic colonizers of the bladder, in which cases antibiotic treatment is discouraged^14^. The prevalence of subclinical bacteriuria in pigs, however, is not accounted for and arguably could be a rare condition considering that the condition, in humans, is mainly associated with the elderly or patients with compromised bladder-voiding ability^14^. This is further supported by our experience using pigs as experimental model of UTI, in which spontaneous bacteriuria in pigs arriving directly from a conventional herd is very rare (less than 1 in a 100, unpublished data)^9–12,15^.

Previously, positive nitrite or blood in urine dipstick tests have been considered positive for porcine UTI, which is congruent with our findings demonstrating a positive predictive value of 100% for both of these parameters^7^. However, we found that blood was negative in most animals with UTI (6 of 9) suggesting that this parameter is unreliable to rule out *E. coli* UTI. All three animals with detectable blood had severe inflammation based on histopathological examinations. This finding suggests that the sensitivity of the dipstick test is associated with the level of inflammation.

Leucocytes were only positive in 3 of 9 animals with infection when using the Combur 7 test. Similar to the blood parameter, detectable leucocytes were only seen in animals with severe inflammation based on histopathological examination. The Multistix 5 was negative for leucocytes in all cases. These discrepancies between tests of different manufacturers support the need for solid validation. The collection of urine 2 days after inoculation could coincide with attenuation of the acute inflammatory response, thus explaining the low levels or complete absence of detectable leucocytes. This is congruent with previous studies showing that the acute inflammation attenuates over time in pigs with ongoing UTI, despite persistent bacteriuria^8,9^. Another explanation for non-detection of leucocytes, could be that clean-catch urine specimens were collected as midstream urine, which may contain low levels of leucocytes due to sedimentation of these cells in the bottom of the bladder as previously demonstrated by ultrasonographic imaging^4^.

We chose *E. coli* as model organism since it is the most common etiological agent of porcine UTI accounting for up to 70% of cases^2^. Furthermore, this species is the only reported pathogen compatible with experimental infection in pigs. Using the UTI89 strain, which is originally a human UTI isolate, entails uncertainties regarding the translation of the results to practice in commercial herds. Experimental studies by Ransley and coworkers using porcine *E. coli* isolates have shown similar infectious outcome, i.e., robust, persistent colonization, in pigs with surgically induced reflux^16,17^. Furthermore, studies have shown clonal link between *E. coli* from pork, human feces and patients with UTI suggesting that UTI is a zoonosis^18,19^. In a study by Khan and co-workers, most *E. coli* isolates from pigs belonged to phylogroup B2 which is the most common group of human UTI isolates^20^. In the same study, the porcine group B2 isolates shared many virulence genes with human pathogenic isolates, with over 70% of isolates harboring *fimH*, a canonical virulence factor for human UTI^20^. *FimH* is expressed by UTI89 and, similar to humans, this gene is recently demonstrated to be essential for successful UTI in pigs^21^. Taken together, the similarity in phylogenetic group and virulence factors of human and porcine *E. coli* isolates indicate that UTI89 may appropriately reflect the infectious outcome of porcine isolates.

Although the experimental protocol offers significant advantages by using controlled experimental conditions the study also has limitations. Mainly, the use of only a single uropathogenic strain makes it difficult to truly conclude on the practical applicability of these findings in the settings of conventional herds where pigs may be infected by other etiological agents (e.g. *Klebsiella* spp., *Streptococcus* spp., *Staphyloccocus* spp., *Enterococcus* spp.). Also, the use of only 10 animals makes it difficult to make strong conclusions applicable to larger herds. This may also explain why only nitrate was significantly different between baseline samples and after inoculation. Blood, leucocyte, and protein was not significantly different, likely as a result of the small study population. Since this study was part of a tandem study with another primary purpose, we only had one control animal. Having only one pig becoming mock-infected did not allow us to conclude on the influence of the inoculation procedure. Particularly the detection of blood could be a result of urethral- or bladder trauma caused by the catheterization procedure.

In conclusion, this study adds new insight into the diagnostic accuracy of urine dipstick tests during porcine UTI. Dipstick tests that are positive for blood, leucocytes or nitrite are strongly associated with *E. coli* UTI. However, due to a low sensitivity of blood and leucocytes, only nitrite may be recommended as a reliable biomarker in excluding *E. coli* UTI.

## Data Availability

All data generated or analyzed during this study are included in this published article.
